# Common Accidents of Every-Day Life

**Published:** 1887-12-10

**Authors:** Robert A. P. Forrester

**Affiliations:** B.A.Dub., M.B. and C.M.Edin.


### The Common Accidents of Every-day Life.

By Robert A. P. Forrester, B.A.Dub., M.B. and C.M.Edin.

### Bones, Fractures, Dislocations.

The skeleton in the adult consists of two hundred distinct bones, which are divisible into four classes—*long, short, flat, and irregular*. Chemically, bone is composed of an earthy and an animal substance blended together. The earthy portion is mostly phosphate of lime, and the animal matter is chiefly gelatine. The latter imparts toughness to it, and binds the particles of earthy matter together. If you take a long bone, say a rib, and immerse it in a dilute mineral acid for some time, you will obtain the animal matter. The bone will retain its size and shape, but it has become flexible, and it is possible to tie it in a knot. On examining a transverse section of bone with the naked eye, it will be seen to consist of two kinds of tissue. The outer part is dense and compact like ivory, and forms a hard external shell. The interior is less firm, and is made up of thin delicate plates or bars, intersecting each other at various angles, and forming a lattice-like arrangement, and for this reason called *cancellous tissue*. In the interior of long bones, like the thigh bone, there is a hollow canal containing the marrow. Examine the surface still closer with a pocket lens, and numerous minute orifices will be perceived; these are the openings of what are called the *Haversian canals*, which penetrate the entire substance of the *compact tissue*, and contain small blood vessels. These blood vessels nourish the bone. Each bone, except at the articular surfaces, which are covered with cartilage, is invested with a thick fibrous membrane called the *periosteum*. Surgeons are always careful in operations for removing bone for disease to preserve this membrane, as it is from it that new bone is produced. If the periosteum be stripped off in a living bone small bleeding points are seen; these mark the entrance of the blood vessels into the *Haversian canals*. During an amputation, after the saw has been used, the cut surface of the bone will be seen to bleed freely, the blood exuding from the vessels in the same canals. In every bone there is at least one large orifice quite visible to the naked eye, called the *nutrient foramen, i.e.*, the space through which the nourishing artery passes to the marrow. This artery is generally accompanied by one or two veins. Bone is well supplied with nerves.

Bone once formed does not remain during life, but is constantly disappearing and being as constantly replaced. The growth occurs by additions to its free edges and surface. In the skull, for instance, it grows in thickness on its surface and in breadth at the edges. The bones of the limbs grow in two ways. The cartilage or gristle of which it first consists grows at the ends until the bone has reached its full size, and there remains to the end of life as *articular* or *joint cartilage*. But in the centre of the bone the cartilage does not grow with the increase in size of the bone, but becomes coated with successive layers of bone produced by the ossification of the periosteum which lies nearest to the cartilage. In removing a piece of dead bone, say, from the leg, the reason will now be evident why the surgeon is careful to preserve this membrane, if any remain, for it is from it that he expects new bone will be formed to fill up the gap that has been made. For practical purposes it will be useful to remember first the investing membrane, or periosteum, and its blood vessels, then the bone itself composed of two layers of tissue, the *compact and the cancellous* with the *Haversian canals* containing blood vessels, then the marrow in the centre, and, finally, the nutrient foramen. For further information concerning the minute anatomy of bone, readers may be referred to any standard work on "Human Anatomy." It is difficult to give a correct idea of the arrangement of the facts without the aid of diagrams.

*General Treatment of Fractures.*—For the use of students and nurses, and for emergencies, a few words may be said about treatment. It is hardly necessary to urge the propriety of handling a broken limb gently when examining the position and nature of the fracture; also when removing the clothing, and during the general management of the limb before it is dressed.

Rough handling is not only unnecessary, but actually cruel, and a fertile source of inflammation and gangrene. The man of refined feelings and with a kind heart needs no instruction. Those who are lacking in such qualities had better have chosen some other profession than that of the art of healing. What are the indications for treatment?

To restore the fragments to their place as completely as possible; to keep them in position; and to prevent spasms, inflammation, etc. Whether the fracture be recent or seen only after swelling has occurred, it is the duty of the surgeon to replace the fragments as soon as possible. If attended to at once, so much the better, for the muscles offer less resistance then to the force necessary for *setting* the limb. The *setting* of a broken limb will be referred to in the individual cases of fracture. For the purpose of keeping the fragments in position we employ various kinds of splints and other appliances, such as the weight and pulley, the inclined plane, etc. Splints are made of a great many substances, *e.g.*, wood, lead, zinc, tin, pasteboard, copper, iron, etc. Those which one sees in the surgical instrument makers, curved, polished, and grooved, and constructed with the object of meeting a multitude of indications, often fail in doing so, and sometimes defeat some other indication equally important. Occasionally, however, we may hit upon one which exactly suits the requirements of the surgeon. As he does not carry splints about with him, and as he may not have exactly what he wants at the bedside of the patient, it is as well for him to understand how to avail himself of such materials as may be within his reach. A friend of mine, now in Australia, once asked me to look after a bad case of compound fracture of the tibia for him during his absence from home. He had been called up during the night to attend the accident, and being informed of the nature of the case before leaving his home, he took a couple of "lancets" with him to act as splints in default of anything better. I found that they had done their duty admirably, and kept the bone in good position. A medical man of eminence in London made use of his umbrella as a temporary splint for a fracture of the thigh during the transit of the patient to the hospital. Many things are called into requisition to answer this purpose, *e.g.*, reeds, coarse grass, branches of trees, unbroken wheat straw may be quilted between two pieces of cloth and made to form excellent temporary splints. Cardboard boxes, gutta-percha, and pasteboard are very useful materials, as they can be easily moulded to the shape of the limb. It matters little what kind of splint be employed provided that it fulfils the indications to be met. Some surgeons prefer starch and plaster dressings, but one great disadvantage in using these is that we cannot from time to time examine the state of the limb at the seat of fracture, a very essential point, and, besides, if starch or plaster dressings be applied when there is a swelling they become loose after it has subsided.

